# Cryptococcal Meningitis in an HIV-Negative Pulmonary Tuberculosis Patient: A Case Report

**DOI:** 10.7759/cureus.45900

**Published:** 2023-09-25

**Authors:** Muhammad Arsal Naseem, Muhammad Ahmad Khan, Waqar Ali, Muhammad Danial Malik, Wajeeha Aslam

**Affiliations:** 1 Medicine, Mayo Hospital, Lahore, PAK; 2 Internal Medicine, Mayo Hospital, Lahore, PAK; 3 Internal Medicine, Central Park Medical College and Teaching Hospital, Lahore, PAK; 4 Cardiology, Mayo Hospital, Lahore, PAK

**Keywords:** fluconazole, india ink, amphotericin, tuberculosis, hiv, cryptococcal meningitis

## Abstract

Cryptococcal meningitis represents a severe opportunistic fungal infection primarily observed in individuals with compromised immune systems. It frequently manifests in symptoms like headaches, vomiting, cranial nerve complications, and cognitive alterations. However, it's worth noting that up to 15% of cases may exhibit no discernible central nervous system-related symptoms.

A 70-year-old male, previously diagnosed with pulmonary tuberculosis and undergoing treatment with anti-tubercular medications, was admitted due to changes in consciousness, sporadic low-grade fever, and cognitive impairment. An in-depth investigation revealed his HIV-negative and non-diabetic status, as well as his preserved immune competence. A plain CT head showed a communicating hydrocephalus and a lumbar puncture was positive for Cryptococcus neoformans.

Treatment commenced with an induction regimen encompassing amphotericin and fluconazole, concurrently maintaining the anti-tubercular treatment course. The patient's condition displayed improvement, leading to a transition to a maintenance dosage of fluconazole.

This case highlighted an extraordinary occurrence of Cryptococcal meningitis in an HIV-negative patient with no history of immunosuppressant use. Notably, Cryptococcal infection should be regarded as a primary consideration in patients afflicted by pulmonary tuberculosis who subsequently present with altered consciousness. The timely identification and proper management of such instances can substantially mitigate the risks of mortality and morbidity associated with this condition.

## Introduction

Cryptococcal meningitis is a fungal infection primarily impacting individuals with compromised immune systems such as those having AIDS, diabetes, taking immunosuppressants, sickle cell disease, and transplant patients [1-7]. Symptoms encompass conventional signs of meningeal involvement, such as headaches and neck stiffness, alongside systemic indications of widespread afflictions like pneumonia and dermatological manifestations. A lumbar puncture can be diagnostic. Patients can have elevated opening pressure, high protein, and low glucose. The presence of organisms can be confirmed via India ink staining or identification of cryptococcal latex antigen in cerebrospinal fluid (CSF).

Although immunosuppression is commonly linked with cryptococcal infection, instances of cryptococcal meningitis in immune-competent patients have been documented [8]. Despite advancements in diagnostic and therapeutic approaches, acute cryptococcal CNS infections persistently carry substantial mortality rates, affecting hundreds of thousands yearly, irrespective of their accessibility to diagnostic resources or treatment avenues [9].

## Case presentation

A 70-year-old male from Lahore, Pakistan, presented to the Emergency and Resuscitation department with an altered state of consciousness for the past two days. Four months ago, he was diagnosed with sputum-positive pulmonary tuberculosis and started on isoniazid, rifampicin, ethambutol, and pyrazinamide. His fever had subsided, his cough improved, and he had been compliant with treatment. However, he did not undergo repeat sputum testing after initiating therapy. Over the last two days, the patient developed a gradual, progressive altered sensorium accompanied by intermittent low-grade fever, recorded at 100°F. He experienced no weakness or seizures, and there was no photophobia. There was no history of substance abuse, recent travel, or head trauma. Additionally, there was no history of diabetes mellitus, intake of immunosuppressant drugs, liver or kidney disease, previous psychiatric illness, neurological surgeries, or family history of neurological disorders. He had received all recommended vaccinations for his age.

On physical examination, the febrile patient (100.2°F) exhibited tachycardia (108 beats/min), maintained oxygen saturation with room air, and displayed normal blood pressure (110/70 mmHg) and blood glucose levels (132 mg/dl). His Glasgow Coma Scale (GCS) score was 9/15, and Kernig and Brudzinski signs were positive. No focal neurological deficits were noted, and there were no rashes. Bilateral coarse crepitations were heard upon respiratory auscultation. To rule out metabolic causes of altered sensorium, serum electrolytes, liver and renal function tests were performed (Table 1) and an abdominal ultrasound was conducted, all of which showed no abnormalities. Arterial blood gas analysis ruled out hypercapnia.

**Table 1:** Baseline investigations

Laboratory Tests	Patient Values	Reference Range
Hemoglobin	12 g/dL	13-17g/dL
White Blood Cells	14*10^3/ul	4-11 *10^3/ul
Lymphocytes	45%	25-50%
Neutrophils	40%	45-75%
Platelets	438*10^3/ul	150-450 *10^3/ul
Alanine Transaminase	36 U/L	0-40 U/L
Aspartate Transaminase	43 U/L	0-35 U/L
Serum Creatinine	0.8 mg/dl	0.6-1.2 mg/dl
Blood Urea	28 mg/dl	15-40 mg/dl
Erythrocyte Sedimentation Rate	59 mm/hour	< 15 mm/hour
Serum Sodium (Na+)	137 mmol/L	135-145 mmol/L
Serum Potassium (K+)	4.6 mmol/L	3.5-5.1 mmol/L
Serum Calcium (Ca++)	10.4 mg/dl	9-11 mg/dl

**Figure 1:**
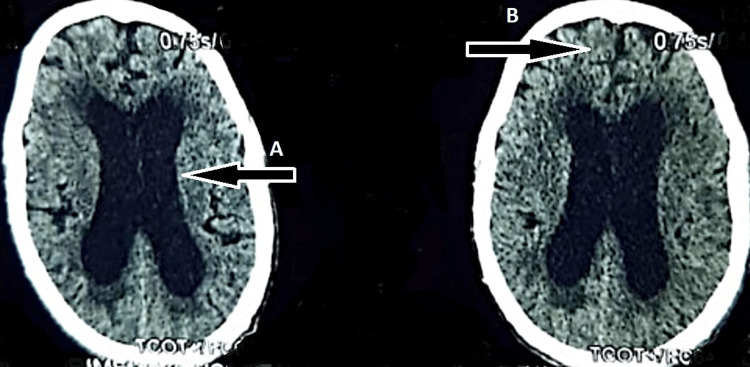
Axial CT brain demonstrating moderate dilatation of the lateral ventricular chain (Arrow A) and cortical atrophic changes mainly involving the bilateral frontotemporal regions in the form of prominent sulci (Arrow B)

A plain computed tomography (CT) scan of the brain (Figure 1) performed in the Emergency department revealed communicating hydrocephalus with dilated third and lateral ventricles.

Our initial suspicion was tuberculous meningitis, and we planned to send a sample for gene Xpert testing to assess for multidrug-resistant tuberculosis (MDR-TB). Following consultations with the Neurology and Neurosurgery units, a Lumbar Puncture (LP) was performed. The results of the Cerebrospinal fluid analysis from the LP are shown in Table 2.

**Table 2:** Cerebrospinal fluid analysis

	Results	Reference Range
Appearance	Clear Watery	Clear
Xanthochromia	Negative	Negative
Opening Pressure	25 cmH2O	10-20 cmH2O
Protein	48.8 mg/dl	20-45 mg/dl
Glucose	< 5 mg/dl	40-74 mg/dl
White Blood Cells	95/uL	< 5/uL
Lymphocytes	80%	0%
Neutrophils	20%	0%
Red Blood Cells	< 5/uL	20/uL
Staining	Yeast cells present	-----
Lactate Dehydrogenase	56 U/L	0-40 U/L
India Ink Stain	Positive	-----

To our surprise, yeast cells were found on staining. It prompted us to send a sample for India ink staining, which was positive for Cryptococcus. Gene Xpert had turned out to be negative, changing our provisional diagnosis altogether.

Given the Cryptococcal infection, a detailed history was taken, and nothing could be found that suggested the immunosuppressed status of the patient. HIV testing was performed, which turned out to be negative. After consultation with an infectious disease specialist, he was commenced on amphotericin and fluconazole for two weeks with the continuation of antituberculous therapy. The patient showed significant improvement from the third day after initiating antifungals and was discharged two weeks after being fully conscious and oriented. He was then continued on fluconazole maintenance dose and is on follow-up.

## Discussion

Cryptococcal meningitis is frequently linked to underlying immunosuppression in patients, which may stem from conditions like an HIV infection, malignancy, transplants, autoimmune disorders, diabetes mellitus, alcoholism, or medication usage [10]. The annual incidence of cryptococcal meningitis is estimated at around 200,000 cases globally, with approximately 3,400 cases occurring each year in the United States alone [9]. Despite its relatively low occurrence, the one-year mortality rate remains high, ranging between 20% and 30%, even with prolonged antifungal therapy [9]. While most cases involve immunocompromised individuals, some instances have been documented in otherwise immunocompetent patients [5-8]. Our elderly patient had no active HIV infection, diabetes mellitus, or ongoing immunosuppressive treatment but did have progressive pulmonary tuberculosis. Initially, tuberculous meningitis was suspected but later ruled out on confirmation of an underlying cryptococcal infection upon staining with India ink. As a result, it can be inferred that the advancement of pulmonary tuberculosis played a role in the development of cryptococcal meningitis [11]. Similar associations between cryptococcal meningitis and tuberculosis have also been observed in HIV-negative individuals [12-14]. For hosts without an underlying compromised immune system or a transplant history, the treatment regimen comprising amphotericin B, fluconazole, and flucytosine may last six to 12 months, involving the induction, consolidation, and maintenance stages [13].

## Conclusions

In immunocompetent patients who present with altered consciousness and a history of pulmonary tuberculosis, cryptococcal infection should be considered. The timely identification and proper management of such cases can significantly reduce the risks of mortality and morbidity associated with this condition.

## References

[1] Poley M, Koubek R, Walsh L, McGillen B (2019). Cryptococcal meningitis in an apparent immunocompetent patient. J Investig Med High Impact Case Rep.

[2] Zhao J, Weng W, Chen C, Zhang J (2021). The prevalence and mortality of cryptococcal meningitis in patients with autoimmune diseases: a systematic review and meta-analysis. Eur J Clin Microbiol Infect Dis.

[3] Sheikh S, Javed U (2022). Cryptococcal meningoencephalitis in an immunocompetent patient. J Coll Physicians Surg Pak.

[4] Gbané-Koné M, Ouali B, Mègne E, Diomandé M, Coulibaly AK, Eti E, Kouakou NM (2015). Cryptococcal meningitis and bone tuberculosis in an immunocompetent: a case [Article in French]. Pan Afr Med J.

[5] Barasa L, Sokwala A, Riunga F, Sokhi DS (2022). A case report of concurrent cryptococcal and tuberculous meningitis in an immunosuppressed renal transplant patient. Cureus.

[6] Acharya R, Khanal K, Upadhyaya P, Kafle S, Savaliya V (2020). Diabetes mellitus as a risk factor for cryptococcal meningitis in immunocompetent. IDCases.

[7] Chau TT, Mai NH, Phu NH (2010). A prospective descriptive study of cryptococcal meningitis in HIV uninfected patients in Vietnam - high prevalence of Cryptococcus neoformans var grubii in the absence of underlying disease. BMC Infect Dis.

[8] Vandroz E, Froidure M, Sifaoui F, Legout L (2019). Cryptococcal meningoencephalitis in an elderly immunocompetent non-HIV-infected patient. Med Mal Infect.

[9] Hardy RE, Cummings C, Thomas F, Harrison D (1986). Cryptococcal pneumonia in a patient with sickle cell disease. Chest.

[10] Jarvis JN, Harrison TS (2007). HIV-associated cryptococcal meningitis. AIDS.

[11] Costa ML, Souza JP, Oliveira Neto AF, Pinto E Silva JL (2009). Cryptococcal meningitis in HIV negative pregnant women: case report and review of literature. Rev Inst Med Trop Sao Paulo.

[12] Molina G, Perozo MA, Mora R, Stratidis J, Freites N (2023). Cryptococcal meningitis in an apparent immunocompetent host. Cureus.

[13] Fang W, Zhang L, Liu J (2017). Tuberculosis/cryptococcosis co-infection in China between 1965 and 2016. Emerg Microbes Infect.

[14] Musabende M, Mukabatsinda C, Riviello ED, Ogbuagu O (2016). Concurrent cryptococcal meningitis and disseminated tuberculosis occurring in an immunocompetent male. BMJ Case Rep.

